# Development of Rabbit Monoclonal Antibodies for Detection of Alpha-Dystroglycan in Normal and Dystrophic Tissue

**DOI:** 10.1371/journal.pone.0097567

**Published:** 2014-05-13

**Authors:** Marisa J. Fortunato, Charlotte E. Ball, Katrin Hollinger, Niraj B. Patel, Jill N. Modi, Vedika Rajasekaran, Dan J. Nonneman, Jason W. Ross, Eileen J. Kennedy, Joshua T. Selsby, Aaron M. Beedle

**Affiliations:** 1 Department of Pharmaceutical and Biomedical Sciences, University of Georgia, Athens, Georgia, United States of America; 2 Center for Undergraduate Research, University of Georgia, Athens, Georgia, United States of America; 3 Department of Animal Science, Iowa State University, Ames, Iowa, United States of America; 4 United States Department of Agriculture Agricultural Research Service, United States Meat Animal Research Center, Clay Center, Nebraska, United States of America; University of Minnesota, United States of America

## Abstract

Alpha-dystroglycan requires a rare *O*-mannose glycan modification to form its binding epitope for extracellular matrix proteins such as laminin. This functional glycan is disrupted in a cohort of muscular dystrophies, the secondary dystroglycanopathies, and is abnormal in some metastatic cancers. The most commonly used reagent for detection of alpha-dystroglycan is mouse monoclonal antibody IIH6, but it requires the functional *O*-mannose structure for recognition. Therefore, the ability to detect alpha-dystroglycan protein in disease states where it lacks the full *O*-mannose glycan has been limited. To overcome this hurdle, rabbit monoclonal antibodies against the alpha-dystroglycan C-terminus were generated. The new antibodies, named 5–2, 29–5, and 45–3, detect alpha-dystroglycan from mouse, rat and pig skeletal muscle by Western blot and immunofluorescence. In a mouse model of fukutin-deficient dystroglycanopathy, all antibodies detected low molecular weight alpha-dystroglycan in disease samples demonstrating a loss of functional glycosylation. Alternately, in a porcine model of Becker muscular dystrophy, relative abundance of alpha-dystroglycan was decreased, consistent with a reduction in expression of the dystrophin-glycoprotein complex in affected muscle. Therefore, these new rabbit monoclonal antibodies are suitable reagents for alpha-dystroglycan core protein detection and will enhance dystroglycan-related studies.

## Introduction

Dystroglycan, a dystrophin-associated glycoprotein, was first isolated from skeletal muscle membranes [1], [2]. Encoded by the gene *DAG1*, dystroglycan is post-translationally cleaved to form two separate proteins, alpha-dystroglycan (αDG) and beta-dystroglycan (βDG), which remain in complex by noncovalent interactions [3]. Dystroglycan has since been found to be expressed in a wide variety of tissues where it plays a number of roles, including involvement in muscle integrity, cell signaling, and development [4], [5], [6]. In healthy skeletal muscle, dystroglycan is located at the sarcolemma as a component of the dystrophin-glycoprotein complex (DGC) [7]. It is through involvement in this complex that dystroglycan forms a structural link between the extracellular matrix and the muscle cell cytoskeleton. αDG is located extracellularly and interacts at the muscle membrane with βDG, a transmembrane protein. αDG provides a structural connection between muscle fibers and the basement membrane by binding to laminin and other extracellular proteins [4]. αDG undergoes extensive post-translational glycosylation, including *O*-mannose glycan structures that are rarely found on mammalian proteins, but are required for functional binding to extracellular matrix proteins [8], [9].

Disruption of dystroglycan is associated with muscle, brain, and other tissue abnormalities. Secondary loss of α- and βDG protein at the muscle membrane occurs in some disorders of the DGC, such as Duchenne and Becker muscular dystrophies [10], [11]. Primary dystroglycanopathies caused by mutations in the *DAG1* gene encoding α- and βDG have been reported in two patients, affecting dystroglycan function by impairing glycosylation of αDG or by presumed disruption of the αDG – βDG binding interface [12], [13]. In addition, aberrant glycosylation and disruption of the *O*-mannose glycan of αDG, due to mutations in glycan processing genes, leads to the development of muscular dystrophies known as secondary dystroglycanopathies [14]. Dystroglycanopathies cause a range of mild to severe pathologies and include diseases such as Fukuyama congenital muscular dystrophy, Walker-Warburg syndrome, and muscle-eye-brain disease [15], [16], [17]. To date, αDG *O*-mannose glycosylation disorders have been linked to mutations in more than a dozen genes, including *FKTN* (fukutin), establishing a prevalent family of autosomal recessive muscular dystrophies [18]. αDG *O*-mannose glycosylation defects have also been observed directly in cancer tissues and are associated with enhanced metastatic potential [19], [20], [21], further emphasizing the urgent need for accurate detection of αDG independent of its glycosylation status.

Currently, the standard reagents used for detection of αDG are IIH6 or VIA4–1. These reagents are mouse monoclonal antibodies that require the functional *O*-mannose epitope for αDG detection. In dystroglycanopathy disease states, however, these antibodies are unable to detect the glycosylation-deficient αDG. The study of αDG in disease has been restricted by access and supply of limited polyclonal reagents against the αDG core protein (e.g. Gt20adg, [2]). Therefore, it is important to develop a reagent that has the ability to detect αDG regardless of glycosylation state and that offers a potentially limitless supply. To meet this need, a rabbit monoclonal antibody against the C-terminal core protein of mouse αDG (aDGct) was generated. The C-terminal core was selected as an ideal antigen because it has the least post-translational processing and was predicted to be free of challenges created by steric hindrance. The rabbit host was selected in an effort to reduce the chance of nonspecific detection in mouse tissue, as many dystroglycanopathies are modeled in mice.

Three rabbit monoclonal antibodies against the core protein of αDG – aDGct 5–2, 29–5, and 45–3– were generated and tested. The ability to specifically detect αDG in mouse, rat, and pig tissue as well as in two models of muscular dystrophy is demonstrated. In addition, these antibodies are capable of detecting both normal and glycosylation-deficient protein species, providing valuable tools for investigating αDG in disease states.

## Materials and Methods

### Ethics Statement

Mice and rats were housed and tissues were collected in accordance with the recommendations of the Guide for the Care and Use of Laboratory Animals; all protocols were approved by the University of Georgia Institutional Animal Care and Use Committee (animal use protocols #A2010 08–153 and #A2013 07–016). All pig procedures were reviewed and approved by the U.S. Meat Animal Research Center Animal Care and Use Committee. All procedures complied with the Guide for the Care and Use of Agricultural Animals in Agricultural Research and Teaching [23]. All efforts were made to minimize animal suffering.

### Animals

The tamoxifen-inducible *Fktn* dystroglycanopathy mouse model has been described previously [22]. Briefly, Cre excision of *Fktn* exon 2 was initiated via two doses of tamoxifen in healthy littermate control (LC) and tamoxifen-inducible knockout (KO) mice by oral gavage. Mice were euthanized by cervical dislocation 2.5 weeks post-tamoxifen and hind limb skeletal muscle was dissected and frozen in liquid nitrogen for biochemical analyses. Alternately, mouse hearts and brains for biochemistry were dissected from a vehicle treated, healthy littermate control and a tamoxifen-treated KO mouse at 21 weeks old (15 weeks post-tamoxifen); brains were flash frozen in liquid nitrogen and hearts were frozen in liquid nitrogen-cooled 2-methylbutane (Sigma). For immunofluorescence, LC and inducible KO Tam-cre/*Fktn* mice were dosed with tamoxifen twice at 6 weeks old and another two times at almost 16 weeks old. Calf muscles were dissected from the mice at 18 weeks old, covered in cryomatrix (ThermoFisher), and frozen in liquid nitrogen-cooled 2-methylbutane (Sigma) for cryosectioning.

Rat skeletal muscle, heart and brain were collected from a young adult male Sprague-Dawley rat (Harlan, Dublin, VA). Briefly, the rat was anesthetized with ketamine/xylazine by intraperitoneal injection and underwent an unrelated surgery for practice intrathecal injection. Following the surgery, the rat was euthanized by cervical dislocation; quadriceps, heart and brain were collected and frozen in liquid nitrogen for biochemistry; tibialis anterior muscle was frozen for immunofluorescence as above.

The Becker muscular dystrophy-affected pig has been described previously [24], [25]. At the time of sacrifice (previously described), skeletal muscle was taken from littermate and dystrophin-insufficient BMD-affected pigs. Diaphragm muscle was frozen for biochemistry and immunofluorescence as above [24], [25].

### Cloning

The sequence encoding the mouse αDG C-terminus (aDGct, mouse amino acids 484–651; Uniprot #Q62165) was amplified from C57BL/6J skeletal muscle cDNA using primers (according to GenBank Accession BC007150, nt 1774–2278, plus start codon, stop codon and modifications for cloning) sense 5′-GACACCATGGGAGTGCCCCGTGGGGGAGAA-3′ and antisense 5′-AAGAATTCAGCCCCGAGTGATGTTCTGAAG-3′ by PCR with Easy A high fidelity enzyme (Agilent, Santa Clara, CA). The purified DNA fragment was inserted into pCR8 (Life Technologies, Grand Island, NY), confirmed by sequencing, then inserted into vector pET29a (EMD-Millipore, Billerica, MA) using NcoI and EcoRI (New England BioLabs, Ipswich, MA). Proper orientation of insertion was confirmed by restriction enzyme digestion and additional Sanger sequencing (Georgia Genomics Facility, Athens, GA).

### Recombinant protein purification and concentration

aDGct-pET29a was transformed into BL21-Gold(DE3) competent *E. coli* (Agilent) and the overnight express autoinduction system 1 (EMD-Millipore) was used to induce protein expression in the presence of 100 µg/ml kanamycin. Bacterial pellets were resuspended in PBS/protease inhibitor buffer (protease inhibitor cocktail: pepstatin A 0.6 µg/ml, aprotinin 0.5 µg/ml, leupeptin 0.5 µg/ml, calpain I inhibitor 2 µM, calpeptin 2 µM, PMSF 0.1 mM, benzamidine 0.75 mM) and were lysed by French cell press. Samples were solubilized in triton X-100 (Fc  = 1%) for 20 min at 4°C before centrifugation at 12,000×g for 10 min. Triton-insoluble pellets were disrupted by sonication in 4 M urea sonication buffer (50 mM Tris pH 7.4, 4 M urea, 0.6 µg pepstatin A, 2 µM calpain I inhibitor, 2 µM calpeptin). Sonicated samples were spun at 4000×g and the resultant supernatants collected.

aDGct sonicated urea supernatants were precleared on DEAE ion exchange resin and resulting DEAE void fractions were mixed with NaCl to reach a final concentration of 100 mM. aDGct samples were then run over S-protein agarose for S-tag protein purification according to the manufacturer's instructions (EMD-Millipore). Protein was eluted from the column in two phases using a total of 3 bed volumes of 0.2 M citrate. Eluent was neutralized by the addition of 1 M Tris pH 8 at 1∶10 dilution and 10 N NaOH at 1∶33 dilution.

Citrate elution fractions were pre-cleared by filtering through 50 kDa cut off spin filters (EMD-Millipore) to remove higher molecular weight background proteins co-purifying with S-tag aDGct. aDGct in the flow through was then buffer exchanged to TBS-G (150 mM NaCl, 50 mM Tris pH 7.4, 1% glycerol) using three rounds of dilution before a final concentration to >1.2 mg/ml using a 10 kDa cut off spin filter (EMD-Millipore).

### Rabbit monoclonal antibody generation

S-tag aDGct antigen was sent to Epitomics, Inc. (Burlingame, CA) for contract custom rabbit monoclonal antibody generation. Two rabbits were immunized with S-tag aDGct recombinant protein in a series of 5 injections using a protocol specifically optimized for the generation of IgG antibodies. Serum from one rabbit had a substantially higher titer by ELISA and detection of endogenous αDG in skeletal muscle lysates; this rabbit was selected for splenectomy and hybridoma fusion according to Epitomics, Inc. technology. Forty-one of 51 multi-clone hybridoma supernatants met minimum requirements for S-tag aDGct antigen detection by ELISA screening and were tested by Western blot on WGA-enriched mouse skeletal muscle and by immunofluorescence on normal and tamoxifen-induced *Fktn* conditional knockout mice according to standard protocols [22]. Multiple clones with the highest specificity and sensitivity were selected for subcloning. Thirty-five subclones were obtained and tested for detection of endogenous mouse αDG. Skeletal muscle lysates from healthy littermates and *Fktn* knockout mice were used instead of WGA-enriched samples to increase the speed and throughput of antibody testing by Western blot. Immunofluorescence was performed as described above. The best subclones were further expanded for Epitomics, Inc. project completion.

### Hybridoma culture

Hybridoma subclones obtained from Epitomics, Inc. were cultured in Epitomics hybridoma growth media (RPMI1640, Epitomics' rabbit hybridoma supplement A, 55 µM 2-mercaptoethanol cell culture grade, fetal bovine serum) according to the manufacturer's instructions. Subclones 5–2, 29–5 and 45–3 were expanded for cryopreservation and supernatant collection. Subclone supernatants were produced in regular serum, low serum, and serum-free conditions according to Epitomics, Inc. protocols. All supernatants were sterile filtered and supplemented with 40 mM Tris pH 7.5 and 5 mM EDTA pH 8 to stabilize solutions for long-term storage. Aliquots of fresh, frozen, and spin concentrated supernatant fractions were tested for each aDGct monoclonal antibody subclone.

### SDS-PAGE and Western blotting

Four hundred micrograms of solubilized skeletal muscle supernatant (in 50 mM Tris, 150 mM NaCl, 1% triton X-100, protease inhibitor cocktail) from TAM-inducible *Fktn* mice, induced knockout (KO) and littermate (LC), were separated on a 3%–15% gradient large-format SDS-PAGE [22]. Four hundred micrograms of skeletal muscle lysate from *Fktn* littermate (healthy) mice was also used as a control for pig and human sample testing. Solubilized skeletal muscle, heart and brain from a Sprague-Dawley rat were prepared in the same manner using 500 µg of solubilized tissue (at concentrations ranging from 6–15 mg/ml) per lane on SDS-PAGE. In addition, total protein lysates of healthy skeletal and cardiac muscle from adult humans were purchased and loaded at 500 µg per lane (BioChain Institute Inc., Newark CA). Proteins were transferred to PVDF and blocked with 1% milk in Tris-buffered saline (50 mM Tris, 150 mM NaCl) plus 0.1% Tween-20. aDGct hybridoma supernatants were tested at a dilution of 1∶5 and detected with goat anti-rabbit HRP secondary antibody at 1∶3000 (Millipore) using the Fluorchem HD2 chemiluminescent imaging system (Protein Simple, Santa Clara CA) with Super Signal West Pico or Dura substrate (Pierce, Rockford, IL). Following aDGct antibody testing, human sample membranes were stripped in mild stripping buffer (1.5% glycine, 0.1% SDS, 1% Tween-20, pH 2.2), rinsed, and reprobed with mouse IgM glycosylated αDG-specific antibody IIH6 at 1∶100 in 1% milk, low salt TBS-T and goat anti-mouse IgM HRP antibody at 1∶3000 (Millipore) [22]. For mouse and rat samples, replicate fresh blots were incubated in antibody IIH6 as above. IIH6 was a kind gift from Dr. Kevin Campbell (University of Iowa).

Skeletal muscle protein, 35 µg per lane, from healthy and dystrophin-insufficient BMD-affected pigs was separated on a 4–20% gradient polyacrylamide mini-gel (Lonza, Rockland, ME). Following separation the protein was transferred to a nitrocellulose membrane (Bio-Rad, Hercules, CA). Membranes were stained with Ponceau S to verify equal loading and transfer. Membranes were blocked with 5% milk in low salt Tris-buffered saline (75 mM NaCl, 50 mM Tris) with 0.1% Tween 20 (low salt TBS-T). Membranes were subsequently incubated with aDGct hybridoma supernatants diluted 1∶3 with 1% milk in low salt TBS–T at 4°C for three days. A secondary donkey anti-rabbit IgG linked to horseradish peroxidase was used at 1∶2000 (GE, Buckinghamshire, UK). ECL reagent (EMD-Millipore) was applied and chemiluminescence was captured with film. Membranes were subsequently stripped for 15 min with restore Western blot stripping buffer (Pierce) and re-probed with 1∶50 IIH6 C4 (Developmental Studies Hybridoma Bank, Iowa City, IA) and 1∶2000 sheep anti-mouse IgM-HRP (GE).

Wheat germ agglutinin (WGA) enrichment was performed on mouse brain and heart lysates, human skeletal muscle and heart lysates, and pig skeletal muscle lysates as described previously [22]. Briefly, mouse and pig tissues were homogenized in 50 mM Tris, 150 mM NaCl, 1% triton X-100, protease inhibitor cocktail and solubilized by rotating at 4°C for 2 hr to overnight. Solubilized supernatants were collected following a 142,000×g spin (or 21,000×g, mouse heart samples only) and protein concentration was determined. Mouse and pig solubilized supernatants and human skeletal muscle and heart lysates were incubated batch method with WGA-agarose (Vector Laboratories, Burlingame CA) overnight at 4°C using 500 µg (for all skeletal muscle and heart samples) or 1 mg (brains only) of starting sample per SDS-PAGE lane. WGA void fractions were collected, WGA agarose beads were washed four times with WGA wash buffer (50 mM Tris, 150 mM NaCl, 0.1% triton X-100, protease inhibitors), and WGA-bound proteins were eluted using 0.3 M *N*-acetylglucosamine in WGA wash buffer.

Rabbit αDG encoding plasmid, DGFc5, with an Fc fusion tag was a gift from Dr. Kevin Campbell (University of Iowa) [26]. HEK-293T cells were transfected with DGFc5 plasmid and cell lysates with recombinant protein were collected and prepared for SDS-PAGE according to standard protocols [27].

### Immunofluorescence

Seven micron cryosections of mouse calf (gastrocnemius and soleus) and rat tibialis anterior were mounted on slides. Tissues were blocked with 5% donkey serum for 30 min at room temperature and then incubated with 5–2, 29–5, or 45–3 aDGct antibody at a dilution of 1∶3 overnight at 4°C. For antigen competition analysis, each monoclonal antibody media supernatant was pre-incubated with 18 µg of purified aDGct protein antigen (50 µL) or 50 µL of PBS for 1.5 hr at 37°C with gentle shaking. Then, 5% donkey serum (in PBS) was added to each pre-mix, the samples were vortexed, cleared by centrifugation, and added to the tissue slide (following the blocking step) for overnight incubation at 4°C. Sections were washed with PBS and incubated with anti-rabbit IgG secondary antibody coupled to Alexa Fluor 546 (1∶500, Life Technologies); along with DAPI nuclear stain (1∶10,000). Slides were mounted using permafluor mountant (ThermoFisher Scientific, Waltham, MA). Fluorescent imaging was performed using an IX71 inverted fluorescent microscope (Olympus, Center Valley, PA). All aDGct antibody images for a particular species were taken under the same conditions. Indirect immunofluorescence for glycosylated αDG using antibody IIH6 was performed as described previously [22]. Images were edited in Photoshop (Adobe) for size, resolution, and intensity; all aDGct images and controls were edited using identical settings.

Ten micron cryosections of pig diaphragm were washed in PBS for 10 min and then blocked with 5% goat serum in PBS at room temperature for 15 min. On each slide one section was incubated overnight at 4°C with antibody 45–3 at a 1∶3 dilution. The second section was used as a negative control and incubated without a primary antibody. The third section was used as a positive control and was incubated with IIH6 at a dilution of 1∶3. The next day, sections were washed in PBS and incubated with secondary antibody. For 45–3, anti-rabbit IgG (H+L) F(ab')2 fragment conjugated to Alexa Fluor 488 (Cell Signaling Technology, Carlsbad, CA) was used at a dilution of 1∶100. For IIH6, anti-mouse fluorescein-conjugated (Millipore, Temecula, CA) was used at a dilution of 1∶100. Slides were mounted with slowfade gold antifade reagent with DAPI (Invitrogen, Carlsbad, CA). All images were taken under the same conditions.

Five micron sections of formalin fixed paraffin embedded human diaphragm from a normal 26 year old male were purchased (Biochain, Newark CA, Cat# T2234169, Lot #B312032). Sections were treated to deparaffinized, retrieve antigen and permeabilize for immunofluorescence as follows: Xylene substitute, 2×5 min; 100% ethanol, 2×5 min; 95% ethanol, 5 min; 80% ethanol, 5 min; 70% ethanol, 5 min; 50% ethanol, 5 min; distilled H_2_O, 2×5 min; antigen retrieval solution (10 mM sodium citrate, 0.05% Tween 20, pH 6.0), 30 min at 95°C, then 20 min cooling to room temperature; antigen retrieval solution, 1 wash; rinse with running ddH_2_O, 5 min; 0.2% Triton-X 100 in PBS, 10 min; PBS washes, 3×5 min; block in 5% donkey serum in PBS, 30 min. Incubation with primary and secondary antibodies, slide mounting and imaging were performed as described for mouse and rat immunofluorescence (see above).

### Image analyses and statistics

The intensity of each αDG protein band on Western blots of pig skeletal muscle lysates was detected using Carestream Molecular Imaging software (Carestream Health, Inc., Rochester, NY). All measurements were adjusted for background signal. The αDG band intensity of each healthy animal sample was averaged to obtain the mean normal αDG intensity for each blot. The αDG band intensity for each healthy and BMD-affected sample was then divided by the average control αDG intensity from the same blot to obtain a measure of the relative αDG quantity in each sample, as detected by the respective antibody. These data were plotted by scatter plot with mean and SEM using Prism 5 (GraphPad, La Jolla, CA). Total protein loaded per lane was quantified from Ponceau S stained blots prior to Western blotting. Protein loading from healthy versus BMD-affected pig samples was not significantly different (two-tailed, unpaired T-test).

For immunofluorescent image quantification, 4 representative images were collected from a section of each animal. Images were converted to a binary format using the density slice function in Openlab (PerkinElmer, Waltham, MA) and the total number of positive pixels determined. Total positive pixels of the 4 representative images were summed for each animal and normalized to the average of total positive pixels from control samples. Data were plotted as described above.

Statistical calculations were performed using Prism 5 (GraphPad). Each control versus affected sample pair for Western blot band intensities and for immunofluorescent pixel intensities were compared by a two-tailed, unpaired T-test. P-values are indicated in figures as follows: *, P = 0.01–0.05, **, P = 0.001–0.01; ***, P = 0.0001–0.001, ****, P = <0.0001.

## Results

### 5–2, 29–5, and 45–3 detect αDG in murine skeletal muscle from normal and fukutin-deficient dystroglycanopathy mice

**Figure 1:**
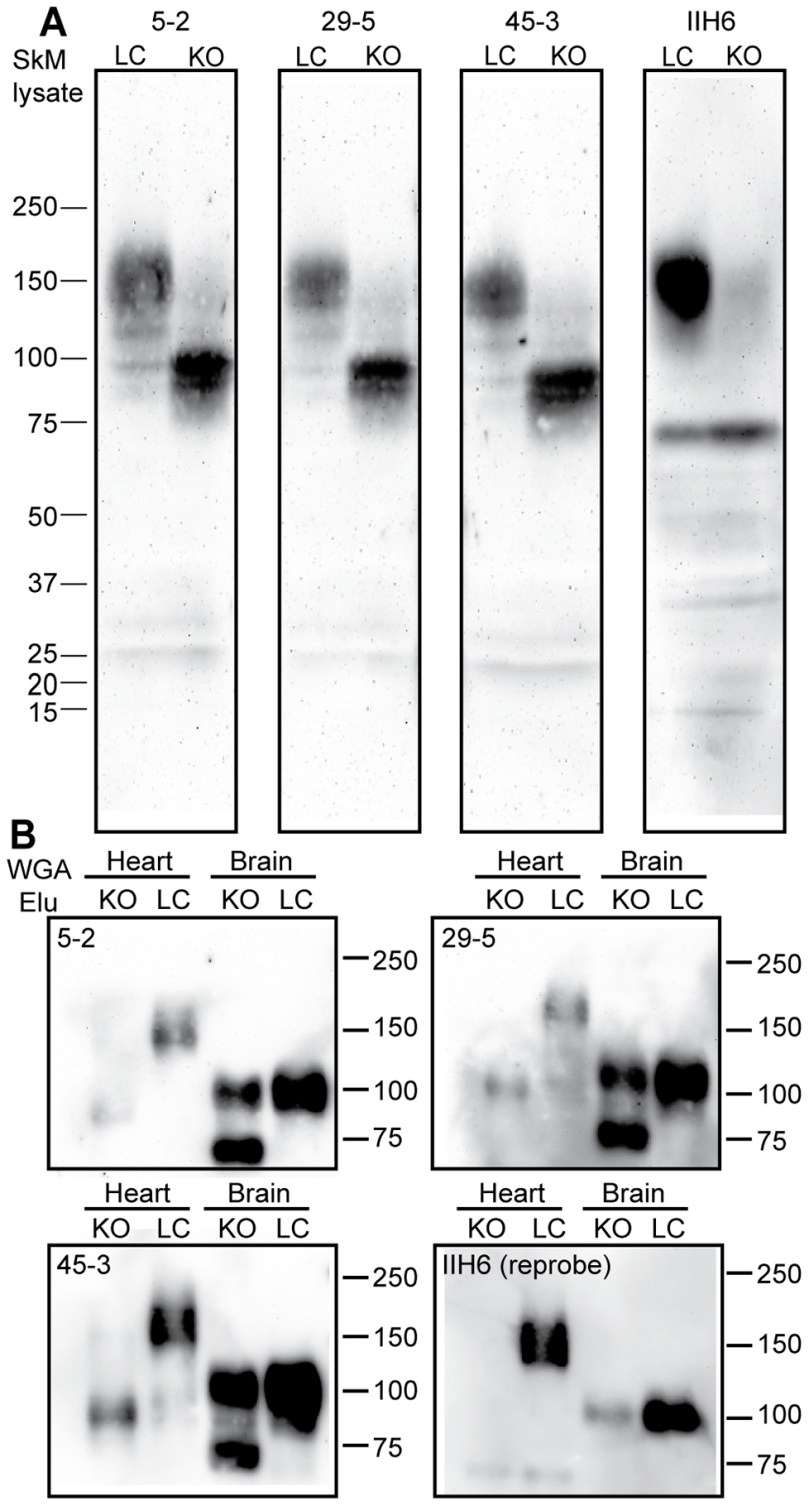
Detection of αDG core protein from skeletal muscle of normal and dystroglycanopathy mice. A) Western blot analysis of αDG core protein detection by rabbit aDGct supernatants. Monoclonal antibodies 5–2, 29–5, and 45–3 were tested on replicate Western blots of solubilized murine skeletal muscle. Lane 1 contains normal murine skeletal muscle (LC) and lane 2 contains skeletal muscle from a mouse with a tamoxifen-induced fukutin-deficient dystroglycanopathy (KO). Detection with antibody IIH6 shows glycosylated αDG for comparison. Molecular weight standards are indicated in kDa. B) Wheat germ agglutinin (WGA) purifications of LC and KO mouse brain and heart lysates were conducted and the elution fraction was analyzed by Western blot. Monoclonal antibody media supernatants 5–2, 29–5, and 45–3 were used to detect αDG core protein on replicate blots; detection with IIH6 was performed by reprobing stripped blots. Molecular weight standards are indicated in kDa.

Rabbit monoclonal antibodies raised against αDG core protein can detect the presence of αDG in skeletal muscle of both normal and dystroglycanopathy mice. Dystroglycanopathies result in abnormally glycosylated αDG, which separates as a lower molecular weight band on SDS-PAGE. To evaluate the ability of the new monoclonal antibodies to detect not only fully glycosylated αDG, but also the smaller abnormal isoform, Western blots were performed on solubilized mouse skeletal muscle lysates from normal littermates and *Fktn*-deficient dystroglycanopathy mice. aDGct antibodies 5–2, 29–5, and 45–3 were each able to detect normal αDG with similar efficacy at dilutions of 1∶5 (Fig. 1A, LC). The specificity of detection was verified by comparison to blots of the same tissue sample labeled with IIH6 (Fig. 1A, far right, LC). This antibody, which detects a glycan epitope of αDG, labeled a broad band of 150 kDa, the same size as the experimental antibodies in healthy mouse skeletal muscle. Importantly, IIH6 detection was greatly reduced or absent in Fktn-deficient skeletal muscle homogenates lacking glycosylated αDG, however these bands are readily apparent in knockout homogenates when exposed to all three aDGct experimental antibodies (Fig. 1A, KO). 5–2, 29–5, and 45–3 each detected a single αDG band at approximately 95 kDa in knockout muscle, indicating reduced glycosylation compared to the unaffected skeletal muscle sample (Fig. 1A, KO).

αDG undergoes tissue-specific glycosylation so its molecular weight varies according to tissue type. To test the sensitivity of aDGct monoclonal antibodies for other αDG species, each media supernatant fraction was used for immunoblot of WGA-enriched proteins from mouse heart and brain. 5–2, 29–5, and 45–3 each detected a high molecular weight band in heart from a healthy mouse that was also labelled by IIH6, confirming its identity as αDG (Fig. 1B, LC). Similarly, all aDGct monoclonal antibodies and IIH6 detected brain αDG at 100 kDa (Fig. 1B, LC). In tamoxifen-treated inducible *Fktn* knockout heart, aDGct monoclonal antibodies detected a band near 95 kDa, consistent with glycosylation-deficient αDG (Fig. 1B, KO). In the brain, 5–2, 29–5, and 45–3 detected two bands, one matching normal αDG and one, near 75 kDa, consistent with glycosylation-deficient αDG in the brain (Fig. 1B, KO). The presence of both bands indicates that tamoxifen-mediated *Fktn* excision was incomplete in the brain of the induced knockout mouse.

**Figure 2:**
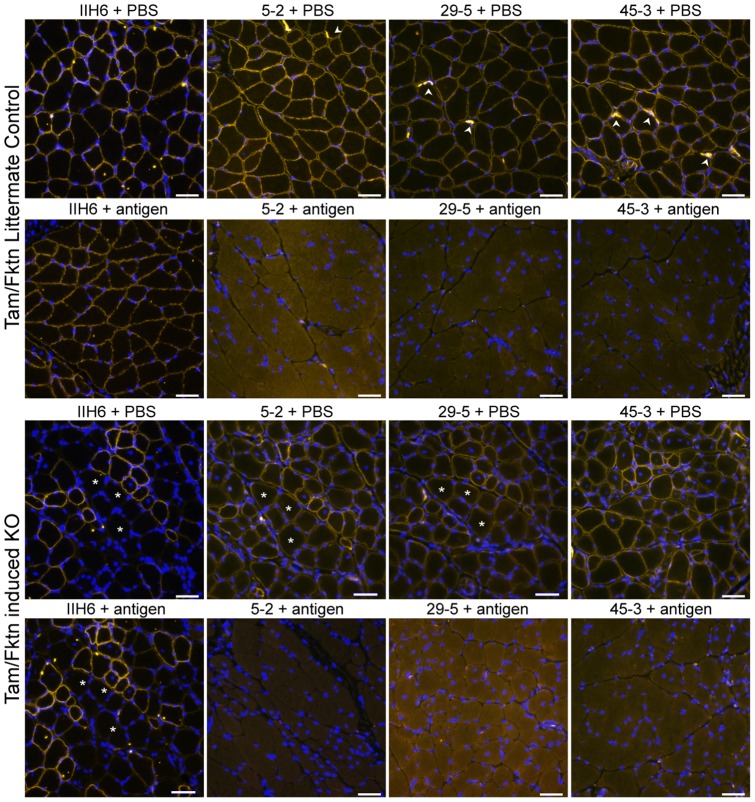
Immunofluorescent detection of αDG core protein on muscle sections of normal and dystroglycanopathy mice in the presence and absence of the aDGct antigen. Calf muscle cryosections from a normal mouse (LC) and a tamoxifen-induced fukutin-deficient dystroglycanopathy mouse (KO) were stained by αDG core monoclonal antibody media supernatants 5–2, 29–5, and 45–3, as well as IIH6, for detection of αDG protein and functional glycan, respectively. Each antibody was pre-incubated with 18 µg of purified aDGct protein antigen or PBS prior to staining. Asterisks mark matching fibers for comparison. Arrowheads mark neuromuscular junctions. 40X objective; 20 µm scale bar.

In immunofluorescent staining of healthy mouse calf muscle (Fig. 2), αDG could be detected by the aDGct 5–2, 29–5 and 45–3 antibodies. There was distinct sarcolemma staining and neuromuscular junctions were strongly labeled with each aDGct media supernatant tested. Notably, pre-incubation of antibody with the aDGct protein antigen blocked labelling, indicating that antibody detection is specific for αDG. Glycosylation-specific staining of αDG by IIH6 was similar to the aDGct core protein antibodies in healthy muscle. IIH6 signal was not blocked by the aDGct protein antigen as it targets a different (glycan) epitope. As expected for core αDG detection, each antibody also stained the sarcolemma of *Fktn*-deficient dystroglycanopathy mouse muscle (Fig. 2 KO). There was some variation in signal intensity that was not strictly correlated with the presence or absence of the αDG functional glycan, shown by IIH6 (Fig. 2 KO). αDG core antibody 45–3 demonstrated the most consistent membrane staining in these experiments. Overall, these data provide evidence of specific recognition of endogenous mouse αDG by aDGct rabbit monoclonal antibodies 5–2, 29–5 and 45–3.

In addition to regular hybridoma subclone media supernatants (regular serum), low serum and serum-free supernatants, with or without spin concentration, were also collected and tested on solubilized murine skeletal muscle from both healthy and dystroglycanopathy mice. All fractions successfully detected both normal and glycosylation-deficient murine αDG by Western blot and immunofluorescence (data not shown). For serum-free and concentrated fractions, Western blot detection was effective at dilutions ranging from 1∶100–1∶1000 (data not shown). Both serum-free and serum-containing monoclonal antibody supernatants also retained αDG detection after one freeze/thaw cycle without the addition of glycerol or other stabilizing agents. For aDGct monoclonal antibody supernatants stored long-term at 4°C, a modest decrease in activity in some samples after 7–8 months of storage has been noted (data not shown).

### 5–2, 29–5, and 45–3 detect αDG in skeletal muscle, cardiac tissue, and brain tissue derived from normal Sprague-Dawley rat

**Figure 3:**
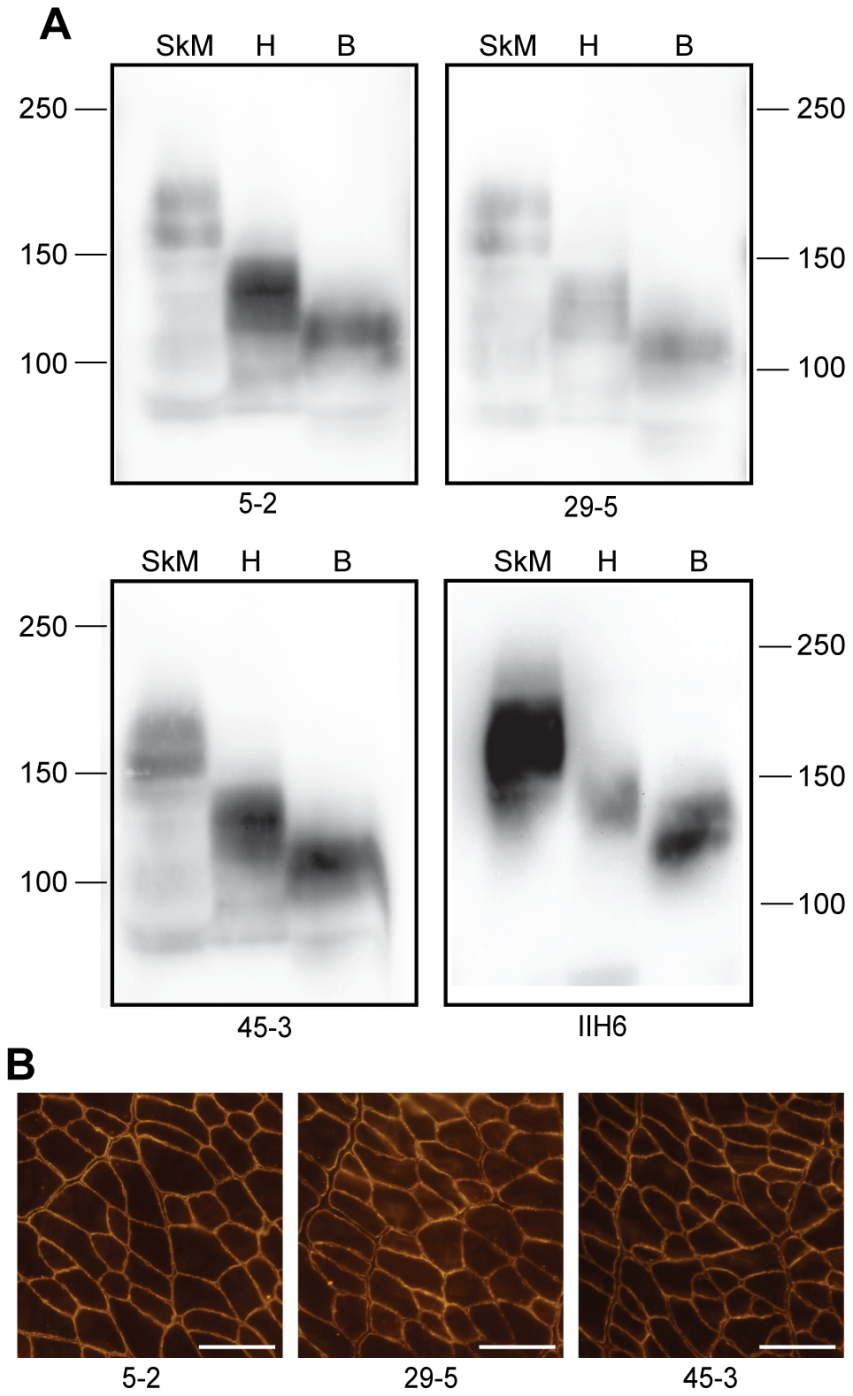
Detection of αDG core protein in rat skeletal muscle, heart, and brain tissue. Detection of rat αDG by aDGct antibody supernatants was analyzed via Western blot and immunofluorescence. A) Monoclonal antibodies 5–2, 29–5, and 45–3 were tested on replicate Western blots of solubilized rat skeletal muscle (lane 1, SkM), rat heart tissue (lane 2, H), and rat brain tissue (lane 3, B). Detection by IIH6 for glycosylated αDG is shown for comparison. Molecular weight standards are indicated in kDa. B) Immunofluorescent detection of αDG in rat tibialis anterior muscle cryosections using antibodies 5–2, 29–5, and 45–3. 20X objective; 100 µm scale bar.

Following successful detection of αDG in mouse, similar experiments were conducted to assess the ability of the experimental aDGct antibodies to detect αDG in tissue derived from rats. Each antibody was used to probe membranes containing solubilized skeletal muscle, heart, and brain taken from a Sprague-Dawley rat. All three antibodies could detect a specific band corresponding to αDG (Fig. 3A). The molecular weight of the αDG detected was variable based on the tissue type (skeletal muscle, heart, or brain), as αDG is known to exist at different sizes in different tissues due to variable glycosylation status [28]. Detection of glycosylated αDG using mouse monoclonal IIH6 revealed bands of corresponding sizes, confirming the detection of αDG by aDGct antibody supernatants. Use of these antibodies to detect αDG on cryosectioned rat tibialis anterior muscle tissue showed positive immunofluorescent staining at the sarcolemma, as expected (Fig. 3B).

### Detection of αDG in a pig model of Becker muscular dystrophy

**Figure 4:**
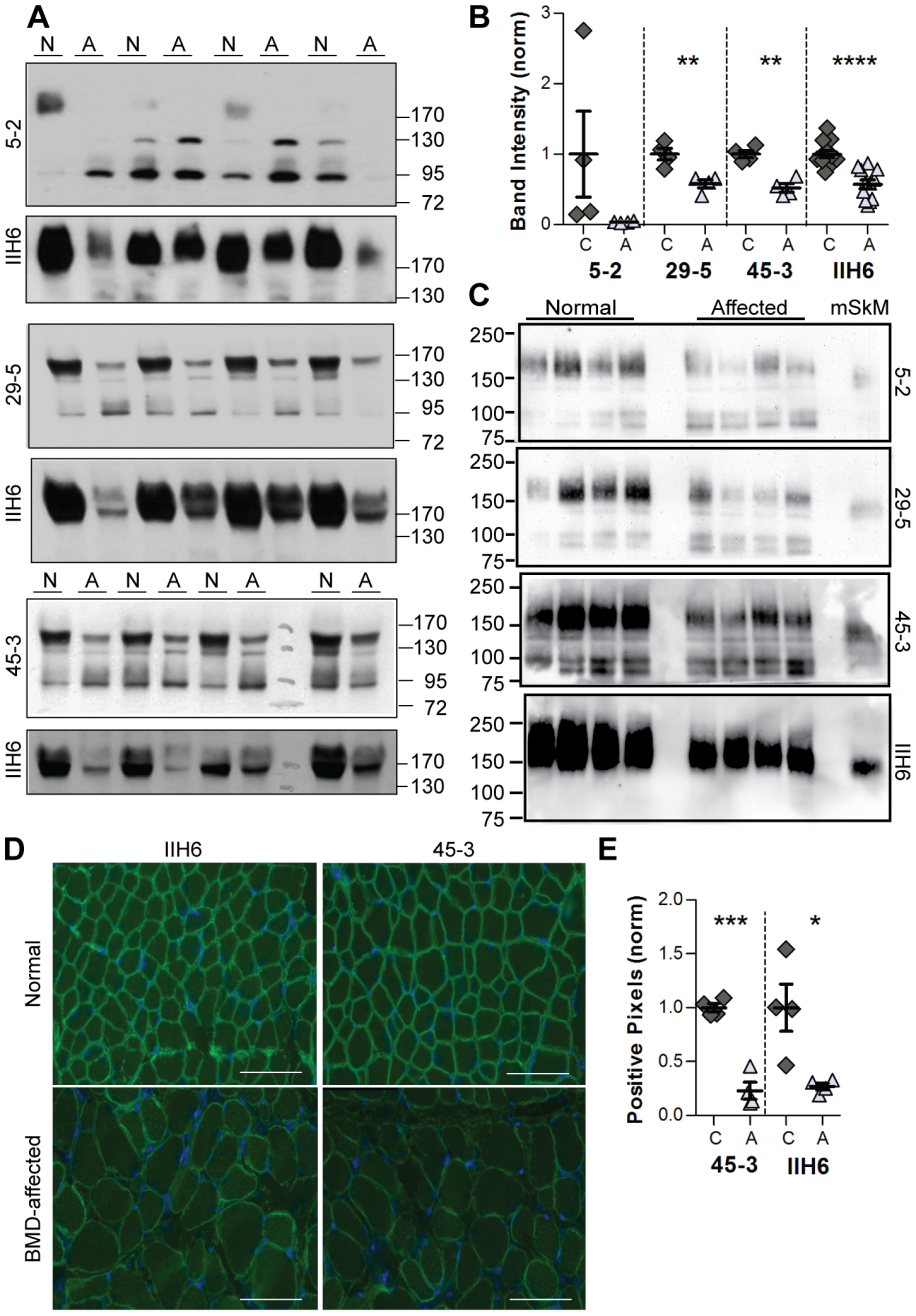
Detection of αDG core protein in a porcine model of Becker muscular dystrophy. A) Immunoblotting of healthy (N) and BMD-affected (A) pig skeletal muscle homogenates was conducted with monoclonal antibodies 5–2, 29–5, and 45–3. IIH6 detection of glycosylated αDG (reprobe) is shown. Molecular weight standards are indicated in kDa. B) Quantification of signal intensity of αDG detection by Western blot. αDG bands were analyzed; the signal intensity for each sample was normalized to the average αDG expression of all controls on the same blot. The resulting relative αDG expression is plotted for each individual control and BMD-affected sample (with group mean and SEM). C) Immunoblotting of WGA-enriched protein eluted from 500 µg of skeletal muscle lysate from normal and BMD-affected pigs using monoclonal antibody media supernatants 5–2, 29–5, 45–3, and IIH6 on replicate blots. Four hundred micrograms of skeletal muscle lysate (mSkM) from a normal mouse was run along with the pig WGA elutions for comparison. D) Diaphragm muscle from healthy (normal) and BMD-affected pigs (BMD-affected) were stained with 45–3 and IIH6. 20X objective; 100 µm scale bar. E) Quantification of pig αDG in normal and BMD-affected muscle by immunofluorescence. The total number of αDG positive pixels summed from 4 representative images was normalized to the average of all normal images per antibody for each control and BMD-affected sample. Asterisks indicate statistical significance between normal and affected pairs; * P = 0.01–0.05, ** P = 0.001–0.01, *** P = 0.0001–0.001, **** P = <0.0001.

After determining that aDGct antibodies 5–2, 29–5, and 49–3 can detect αDG in both its normal and abnormally glycosylated states in rodent tissues, the antibodies were tested for the ability to detect αDG in an emerging disease model species, when abundance of the protein is significantly reduced. Becker muscular dystrophy (BMD) is a condition in which disruption in the dystrophin gene and the subsequent decrease in cytoplasmic dystrophin cause α- and βDG accumulation to be reduced at the sarcolemma. IIH6 detection of glycosylated αDG on BMD-affected pig skeletal muscle revealed a significantly fainter band of equivalent molecular weight compared to the control pig skeletal muscle, therefore showing a reduction in the amount of αDG present, but not its size (Fig. 4A, B, IIH6). Interestingly, αDG detection appeared to arise from a doublet of bands at 170 and 150 kDa in both healthy and BMD-affected samples. When rabbit aDGct antibody supernatants were used, αDG core protein expression was also reduced in BMD-affected samples compared to healthy controls (Fig. 4A, B). aDGct supernatant 5–2 had the weakest detection of αDG, with bands in most but not all the control samples and no detection of αDG in the BMD-affected muscle. Of the rabbit monoclonal antibodies, supernatant 5–2 also detected the highest MW αDG species at 170 kDa (Fig. 4A). An additional 130 kDa band was detected in some samples by 5–2, however this band is expected to be non-specific background as its intensities were not significantly different between BMD-affected and healthy samples. Both 29–5 and 45–3 aDGct antibodies reproducibly detected higher expression of αDG in the control compared to the BMD-affected samples, although they detected the smaller molecular weight population of glycosylated αDG at approximately 150 kDa, suggesting that the antigen epitopes of 29–5 and 45–3 may be more similar than that of aDGct antibody 5–2 (Fig. 4A, B).

To further address the molecular weight of αDG species and potential background detection in pig, skeletal muscle from both normal healthy and BMD-affected pigs was solubilized and enriched by WGA purification. As shown in Fig. 4C, the previous 150 and 170 kDa doublet pattern was replaced by a broad αDG band that was similar to detection of glycosylated αDG by IIH6. Additional bands under 100 kDa were still present at varying intensities that were not correlated with the expression level of the normal αDG protein.

Rabbit media supernatant 45–3 was also tested on pig muscle cryosections because it had the most robust detection on mouse dystrophic muscle sections and on preliminary immunoblot testing with pig lysates. The sarcolemma was labeled with an intensity equivalent to labeling with IIH6 on control pig skeletal muscle sections. Staining of BMD-affected pig sections was again equivalent to that of IIH6, with reductions in sarcolemma staining and patchy fiber labeling as expected in the BMD-affected model (Fig. 3D). A decrease in αDG staining (both core and glycosylated) in BMD-affected muscle sections compared to healthy controls was confirmed by pixel quantification (Fig. 3E).

### Detection of αDG in other species

aDGct monoclonal antibodies were tested on rabbit αDG by Western blot. No major bands were detected in HEK-293T lysates expressing the recombinant full-length rabbit αDG fusion protein DGFc5 (data not shown). The lack of specificity for rabbit protein may be due to the generation of the hybridomas from a rabbit host (although rabbit αDG has 163/168 amino acid identity with the mouse antigen) or steric hindrance by the C-terminal Fc fusion protein.

In addition, both Western blot and immunofluorescence were performed on human samples. For human skeletal muscle and human heart protein lysates, media supernatants of aDGct antibodies 5–2, 29–5, and 45–3 did not detect endogenous human αDG, even though robust αDG signal was detected in both tissues using the glycosylated αDG antibody IIH6 (Fig. S1A). Similar experiments were performed using WGA to enrich αDG (and other glycosylated proteins) from human skeletal muscle and heart (Fig. S1B). While faint bands were detected in WGA void samples by media supernatants of 29–5 and 45–3, there was no detection of endogenous αDG in the WGA elution, which was strongly labelled by IIH6. Detection of αDG from mouse skeletal muscle lysate confirmed that both 29–5 and 45–3 antibodies were active. Antibody 5–2 was not sensitive enough to detect any bands, even mouse skeletal muscle lysate, on the stripped human blots.

The dilute media supernatant antibody fractions used may simply lack the sensitivity to detect human αDG protein. Therefore, serum-free fractions, which contain much higher concentrations of antibody, were also tested (Fig. S1C). At a dilution of 1∶100, serum-free aDGct antibodies 5–2, 29–5, and 45–3 clearly labelled mouse protein but did not detect WGA-enriched human αDG, even though IIH6 showed very strong detection of the glycosylated protein. The serum-free fraction of 45–3, the strongest antibody in these experiments, was further tested at a dilution of 1∶25, but no major human bands were labelled.

Lastly, aDGct monoclonal 5–2, 29–5, and 45–3 media supernatants (at 1∶3 dilution) and serum-free fractions (at dilutions of 1∶20 and 1∶10 for all antibodies, and 1∶5, 45–3 only) were tested on formalin fixed paraffin embedded human diaphragm sections using indirect immunofluorescence (Fig. S1D and data not shown). The muscle fiber membranes were clearly labelled by glycosylation-specific antibody IIH6. However, 5–2, 29–5, and 45–3 treated sections showed only occasional puncta and rare short regions of muscle membrane staining that were similar to background staining with no primary antibody. Consequently, antibodies 5–2, 29–5, and 45–3 appear to have low or no sensitivity for human αDG.

## Discussion

To date, research into the role of dystroglycan glycosylation in various pathological processes has been restricted by the limited supply of suitable reagents for detection of abnormally glycosylated αDG. The standard reagent for αDG detection, IIH6, cannot distinguish between an absence of αDG protein versus a failure in functional glycosylation of αDG due to its requirement of the functional *O*-mannose glycan for αDG detection. The most commonly used reagents for non-glycan-dependent αDG detection have been polyclonal antisera Gt20adg, Sheep 5, and sheep anti-αDG [2], [29]. Such reagents, however, are limited in supply and are not commercially available. Pavoni et al. generated a mouse monoclonal antibody against a highly conserved sequence in the αDG C-terminus (Fig. 4, amino acids 547–564); however the antibody was unable to detect fully glycosylated αDG, limiting its application [30]. While detection of βDG is often used as a proxy for dystroglycan protein expression, it is not ideal since αDG can be shed from the dystroglycan complex, particularly in cell culture conditions [31]. The monoclonal antibodies generated in this study provide a much improved alternative, as they can easily be generated from hybridoma subclones, they are more specific in epitope recognition than polyclonal sera, and they can directly detect αDG.

Tests with immunoblotting and immunofluorescence show that rabbit aDGct supernatants 5–2, 29–5 and 45–3 are relatively similar in the ability to detect αDG core protein in skeletal muscle, heart, and brain of mouse and rat, although in side-by-side experiments signal from 45–3 tends to be a little stronger. Detection in pig tissue had more variability, with 5–2 showing more limited ability to label αDG than 29–5 and 45–3. While glycosylated αDG typically distributes as a broad band, indicating a mixture of variably glycosylated subspecies, it was notable that in pig skeletal muscle lysates, αDG distributed into two more distinct subspecies in most samples detected by glycosylated αDG antibody IIH6. Supernatant 5–2 showed specificity for the higher 170 kDa pig lysate αDG. In contrast, 29–5 and 45–3 only detected the 150 kDa αDG isoform of the lysate pair, but also exposed some additional lower molecular weight bands. The lower molecular weight bands are expected to be nonspecific background rather than αDG degradation products because they did not colabel with IIH6 and there was no apparent pattern of intensities between BMD-affected versus healthy samples.

To further address the multiple αDG species versus potential background bands in pig lysate samples, WGA-enriched samples were also tested. In WGA elution fractions, the distinct αDG doublet found in some lysate samples was no longer apparent. Loss of the αDG doublet could be a technical artifact of two different SDS-PAGE systems (mini-gel vs. large gel). Alternately, unidentified proteins that do not bind WGA could be co-migrating with αDG in lysate, but not WGA elution samples, shifting its distribution. Core αDG monoclonal antibodies showed detection of a broad 145 kDa –190 kDa band, similar to glycosylation-specific IIH6. Additional lower molecular weight bands were also detected by the aDGct antibodies, as in the lysate samples. As the intensity of these bands was not highly correlated, positively or negatively, with intensity of the known αDG band or the sample group (normal vs. affected), it is unlikely that they are αDG protein. However, the possibility of lower molecular weight αDG species with reduced glycosylation or proteolytic cleavage cannot be ruled out.

**Figure 5:**
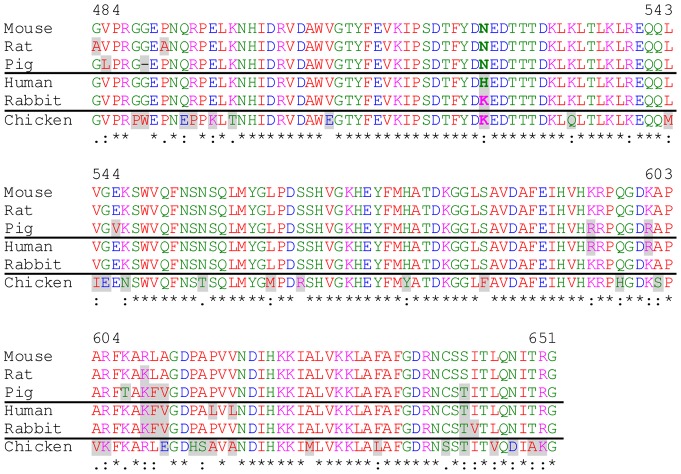
Amino acid sequence alignment of the αDG C-terminus. The mouse αDG C-terminal antigen used for antibody generation is aligned to rat, pig, human, and rabbit species-specific sequences using the Clustal 2.1 multiple sequence alignment tool [31]. The chicken αDG C-terminus is also shown for comparison with a non-mammalian sequence. Amino acid numbering is according to the mouse sequence. Aligned sequences are Uniprot No. Q62165 a.a. 484–651 (mouse), F1M8K0 a.a. 484–651 (rat), I3LD20 a.a. 469–635 (pig), Q14118 a.a. 486–653 (human), Q28685 a.a. 486–653 (rabbit), and A4VAR9 a.a. 487–654 (chicken). Amino acids are colored according to similar properties: red, small; blue, acidic; magenta, basic; green, hydroxyl, sulfhydryl or amine. Consensus across all species aligned is indicated below each amino acid residue by symbols: asterisk, conserved residue; colon, residues with strongly similar properties; period, residues with weakly similar properties; no symbol, no conservation of properties. Each residue that is not identical to the corresponding mouse amino acid is highlighted in gray. Horizontal lines are used to group the aDGct monoclonal antibody detection positive species (mouse, rat, pig) with the aDGct antibody detection negative species (human, rabbit fusion). Asn524 (in bold) is the only residue that is conserved in all detection positive species and not in detection negative species.

Though good detection of αDG was seen among different species – the antibodies were all effective in mouse, rat, and pig – detection of human or recombinant Fc-tagged rabbit αDG was not observed. It is possible that the small number of amino acid differences among species affect the epitopes recognized by 5–2, 29–5 and 45–2. However, comparison of species with aDGct monoclonal antibody detection (mouse, rat, pig) versus species lacking detection in these experiments (rabbit fusion protein, human) only finds one of the 197 amino acid residues of the C-terminal mouse antigen that specifically segregates according to antibody sensitivity (Fig. 5; CLUSTAL 2.1 alignment of Uniprot No: mouse Q62165 a.a. 484–651; rat F1M8K0 a.a. 484–651; pig I3LD20 a.a. 469–635; Human Q14118 a.a. 486–653; rabbit Q28685 a.a. 486–653). Asn524 is shared by mouse, rat and pig, while human and rabbit contain His and Lys residues at the equivalent positions. Therefore, this position or some combination of other non-shared changes might underlie the loss of aDGct antibody sensitivity for detection of human and rabbit species. Lack of detection of rabbit αDG may be due to steric hindrance of the C-terminus by the Fc fusion tag. If that is true, then other residues may contribute to the lack of sensitivity for human αDG. In the antigen region, there are a total of 9 non-identical residues in human versus mouse αDG (Fig. 5; mouse residues 524, 595, 601, 609, 610, 611, 616, 618, and 652). In addition to the Asn524>His difference described above, Pro616>Leu and Val618>Leu are the only other changes in the human sequence that are not shared with the pig. Loss of conformational rigidity with Pro616, in particular, could be important for the antibody epitope.

Dystroglycanopathies result in a loss of αDG functional glycosylation, therefore glycosylation-specific antibody IIH6 can only detect the absence of glycosylated αDG, and not its mutant abnormally glycosylated form. By detecting a core epitope of αDG, these aDGct antibodies fill an important role in that they can provide specific detection of normal and glycosylation-deficient αDG species as demonstrated in fukutin-deficient mice. As the antibodies are monoclonal and produced in rabbit, they are ideally suited for use in mouse dystroglycanopathy models, although other non-*Fktn* dystroglycanopathy mice will need to be tested to validate the antibodies for a wider array of glycosylation-deficient αDG variants. The three aDGct monoclonal antibodies reported here, 5–2, 29–5, and 45–3, may also have some variability in sensitivity for highly glycosylated αDG, as seen in pig skeletal muscle lysates. It is expected that these new core αDG antibodies will be useful in furthering the understanding of dystroglycanopathies and other dystroglycan-linked pathologies.

## Supporting Information

Figure S1 **αDG detection in human tissue samples.** A) Human skeletal muscle (SkM) and heart (H) lysates were analyzed with IIH6 glycosylation-specific αDG antibody and αDG core monoclonal antibody media supernatants 5–2, 29–5, and 45–3 by Western blot. All molecular weight standards are indicated in kDa. B) WGA purification of human SkM and H lysates were conducted. The elution and void fractions from each were immunoblotted with IIH6, and media supernatant fractions of 5–2, 29–5 and 45–3. Skeletal muscle lysate from a normal mouse was included for comparison (Mse SkM). C) WGA purification of human SkM: elution and void fractions, plus normal mouse SkM lysate, were analyzed by immunoblotting with IIH6 and serum-free fractions of monoclonal antibodies 5–2, 29–5, and 45–3. D) Immunofluorescence of formalin fixed, paraffin embedded sections from a normal human diaphragm with IIH6 and serum-free fractions of 5–2, 29–5, and 45–3. Controls with secondary antibody only are shown (2nd ctrl). 20X objective, 50 µm scale bar.
(TIF)
