# Supplementary figures and images for: Development of Rabbit Monoclonal Antibodies for Detection of Alpha-Dystroglycan in Normal and Dystrophic Tissue
Source: PLoS One. 2014 May 13;9(5):e97567. doi: 10.1371/journal.pone.0097567 (PMC4019581; doi:10.1371/journal.pone.0097567)

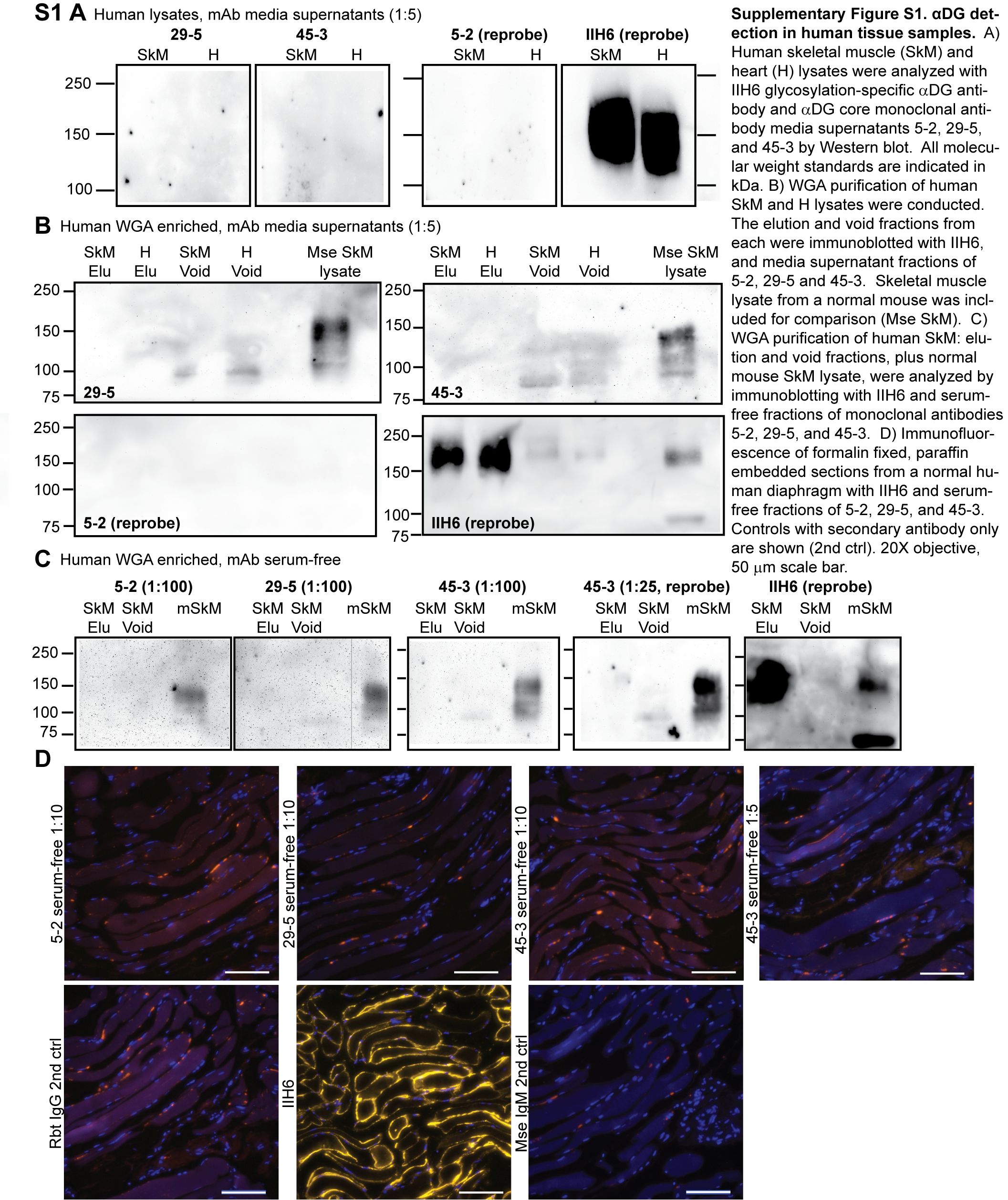

Supplement: Figure S1 — αDG detection in human tissue samples. A) Human skeletal muscle (SkM) and heart (H) lysates were analyzed with IIH6 glycosylation-specific αDG antibody and αDG core monoclonal antibody media supernatants 5–2, 29–5, and 45–3 by Western blot. All molecular weight standards are indicated in kDa. B) WGA purification of human SkM and H lysates were conducted. The elution and void fractions from each were immunoblotted with IIH6, and media supernatant fractions of 5–2, 29–5 and 45–3. Skeletal muscle lysate from a normal mouse was included for comparison (Mse SkM). C) WGA purification of human SkM: elution and void fractions, plus normal mouse SkM lysate, were analyzed by immunoblotting with IIH6 and serum-free fractions of monoclonal antibodies 5–2, 29–5, and 45–3. D) Immunofluorescence of formalin fixed, paraffin embedded sections from a normal human diaphragm with IIH6 and serum-free fractions of 5–2, 29–5, and 45–3. Controls with secondary antibody only are shown (2nd ctrl). 20X objective, 50 µm scale bar. (TIF) [file pone.0097567.s001.tif]
